# Synthesis and characterization of Cu-Doped ZnO nanostructures for UV sensing application

**DOI:** 10.1186/s13065-024-01141-2

**Published:** 2024-02-14

**Authors:** K. Al-heuseen, A. I. Aljameel, R. K. Hussein

**Affiliations:** 1 Department of Applied Science, Al-Balqa Applied University, Amman, Jordan; 2 College of Science, Department of Physics, Imam Mohammad Ibn Saud Islamic University (IMSIU), P.O. Box 90950, 11623 Riyadh, Saudi Arabia

**Keywords:** Electrochemical deposition, Porous Silicon, Cu-doped ZnO, XRD, UV-sensor

## Abstract

In this work, Fabrication, and characterization of Cu-doped ZnO thin films deposited on porous silicon (PSi) substrates have been reported using electrochemical deposition (ECD) technique. The influence of Cu-doping concentrations on morphology, structure, and electrical characteristics of zinc oxide (ZnO) thin films were presented. X-ray diffraction analysis (XRD) has been used to characterize the lattice constants, average size, in-plane (along a-axis) and out of plane (along c-axis) strains for the Cu–ZnO crystals. The effects of Cu-doping concentration on crystal parameters were also investigated from the XRD analysis. The samples were used for UV-sensing applications. In addition, Cu-doped ZnO and pure ZnO metal–semiconductor-metal photodetector, with Cu as electrode contacts were successfully produced for ultraviolet (UV) detection. The *I*-*V* (current–voltage) characteristics were used to study the sensing enhancement. Finally, the UV photodetector based on Cu-doped ZnO films was successfully fabricated and shows a five times enhancement in the sensitivity to UV light compared to that of pure ZnO photodetector.

## Introduction

Zinc oxide (ZnO) thin film materials are known to be the most attractive semiconducting oxides due to their wide optical band gap (about 3.37 eV), large excitonic binding energy (60 meV) at room temperature and high resistivity. The unique characteristics of ZnO made it suitable in wide range of applications including synthesis of laser diodes, optoelectronic device applications, ultraviolet (UV) light emitting diodes, UV detectors and piezoelectric devices [1–3].

The doped/undoped ZnO materials can be fabricated using many techniques such as RF plasmas [4], sputter deposition with ion-assisted cyclic method [5], radio frequency sputter deposition [6], thermal oxidation [7], evaporation with electron beam assistance [8], reactive evaporation [9], spray pyrolysis [10], low pressure metal organic chemical vapor deposition (LPMOCVD) [11], chemical bath deposition [12], and electro chemical deposition [13].

Compared to other techniques, electrochemical deposition (ECD) method offers several advantages over others including fast, simple, low cost, operation at near room temperature, large-scale deposition area. In this method, it is possible to control the films composition and crystalline structure by adjusting the experimental conditions. It is interesting that the electrodeposition of ZnO can also produce a variety of different morphological deposits [14]. In addition, normally, fabrication of ZnO thin films by electrodeposition is considered eco-friendly because of the lack of the toxic chemicals used in the electrolytic bath [14]. Moreover, the deposition process can be carried out on various substrates such as silicon and porous silicon. Porous silicon (PS) has opened new possibilities for Si-based integrated circuits due to its remarkable optical and electronic properties. Applications of PS, including visible photoluminescence at room temperature, highly efficient electroluminescent devices (LEDs), photo detectors, and surface acoustic wave (SAW) devices, have been previously reported [15–17]. Therefore, this technique is well adapted for industrial procedures in fabricating ZnO thin films. Recently, modified ZnO was prepared by doping with different types of metallic ions such as Mg [18], Ti [19], Al [20], In [21], Ag [22], Co [23], Ni and Cu [24–27] to fulfil the needs for various applications. The results of these transition metals doped ZnO show that the optical, magnetic, and electrical properties changed with the change in concentration of metal.

Among various dopants, Cu can be easily doped in the lattice of ZnO for its similar radius and electronic shell to Zn atom. Electronic conductivity of Cu is very high, and it is cheap and highly available on Earth’s crust which makes it an important metal for doping. Many properties of the ZnO thus can be modulated by Cu content [28, 29]. For example, Cu-doping can reduce the band gap and enhance the absorption coefficient [28], which is critical for the application in the UV–visible light region. In addition, Cu significantly affects the electrical, chemical, structural, and optical properties of ZnO, and the study of the electronic state of Cu in ZnO has been the subject of interest for a long time [30–34].

Cu-doped ZnO nanostructures were fabricated in this work by low-cost electrochemical techniques on PSi substrates using an aqueous solution at room temperature. The effect of Cu-doping concentrations on the structural, optical, electrical and the UV-sensing characteristics of ZnO nanostructure were investigated. The composition and structural characteristics were investigated by scanning electron microscopy (SEM) and X-ray diffraction (XRD) techniques.

### Experimental procedures

**Fig.1:**
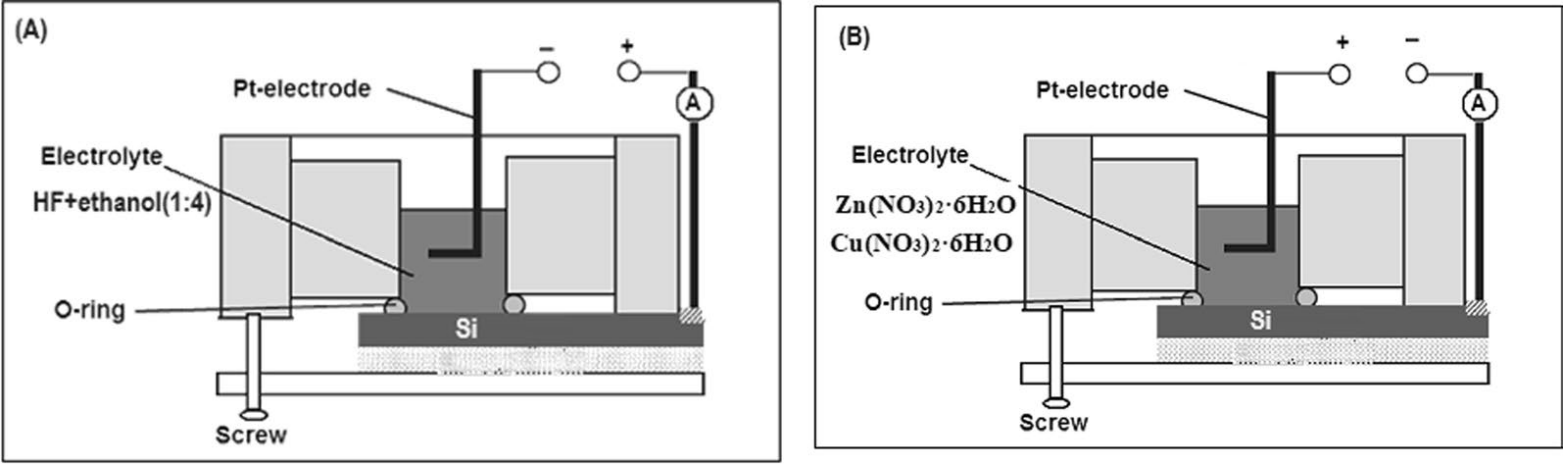
Schematic diagrams of the etching set-up (**A**) and the ECD set-up (**B**) Results and discussions

In the first step the porous n-Si (100) substrate was fabricated by using the electrochemical etching method. A homemade Teflon cell was used, Si (100) sample connected as anode while Pt wire fixed as a cathode, (Fig. 1A) illustrate the set up. A mixture of 49% aqueous HF and 95% ethanol at a ratio of 1:4 by volume were used as an electrolyte, constant current density of J = 30 mA/cm^2^ (supplied by a Keithley 220 programmable current source) was used for 30 min in this step. After etching, the samples were rinsed with deionized water and dried in ambient air. Before the etching process, the substrate was cut into 1 cm^2^ square pieces. The substrate pieces were first cleaned with 50:10:10 (H_2_O:NH_4_OH:H_2_O_2_) for 10 min at room temperature in an ultrasonic bath and then cleaned with 1:50 HF:H_2_O for 10 min and finally cleaned with 60:10:10 (H_2_O:HCl:H_2_O_2_) maintaining the temperature at 80 °C for 10 min more in an ultrasonic bath to remove surface oxides. The substrates were rinsed with deionized water between the cleaning steps.

In the second step Cu-doped ZnO nanostructures were fabricated by one-step electrochemical process using aqueous solution containing Zinc nitrate Zn (NO_3_)_2_·6H_2_O and copper nitrate Cu (NO_3_)_2_·6H_2_O dissolved with deionized water. The electrolytic pH for the solvent was maintained at 3 acidic states with HCl and NaOH and kept at normal atmospheric pressure and room temperature. The films were deposited for different additions of Cu; 5%, 7%, 9%, 11% and 15% wt keeping the molarity of copper nitrate (15µM) fixed. A simple homemade Teflon sensing cell with two electrodes was used for experimental investigations see Fig. (1B). Cu metal contacts were deposited onto portion of the sample using the ECD technique, to fabricate the photodetectors.

## Results and discussions

Zinc nitrate [Zn (NO_3_)_2_·6H_2_O] was used as an electrolytic medium. The deposition potential of ZnO is 0.88 V/NHE which means that the deposition is feasible, thermodynamically, if a cathodic potential of 0.88V or less is applied to the conducting substrate.

The possible electrodeposition mechanisms of ZnO through nitrate precursors can be summarized as follows:

First, reduction of nitrate ions produces nitrite and hydroxide ions in water. Then, zinc ions interact with hydroxide ions forming zinc hydroxides. Following dehydration of Zn (NO_3_)_2_, ZnO is formed as:

1 $$ Zn\left( {NO_{3} } \right)2 + H_{2} O \to Zn^{2 + } + 2NO_{3 - } + H_{2} O $$

2 $$ NO_{3 - } + H_{2} O + 2e^{ - } \to NO_{2 - } + 2OH^{ - } $$

3 $$ Zn^{2 + } + 2OH^{ - } \to Zn\left( {OH} \right)_{2} $$

4 $$ Zn\left( {OH} \right)2 \to ZnO + H_{2} O $$

Reversible reactions in Eqs. (2) and (4) took place due to the much higher solubility of ZnO. This intermediate step involves the formation of hydroxide ions, due to the local increase of pH, allowing the growth under quasi-equilibrium conditions, which subsequently produces high quality ZnO crystals [35].

**Fig. 2:**
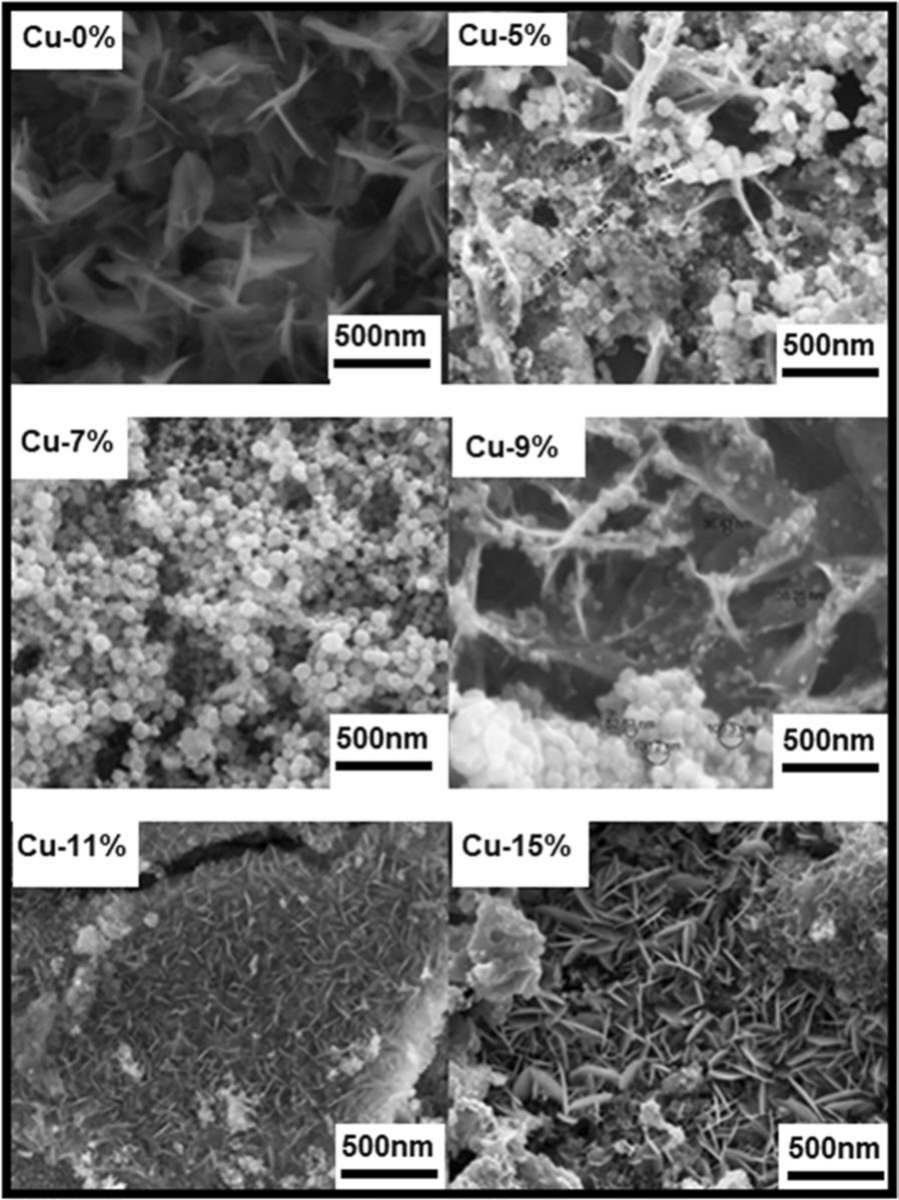
SEM micrographs revealing the surface morphology of ZnO thin films for undoped 0% Cu ZnO and doped with 5% Cu, 7% Cu, 9% Cu, 11% Cu and 15% Cu

**Fig. 3:**
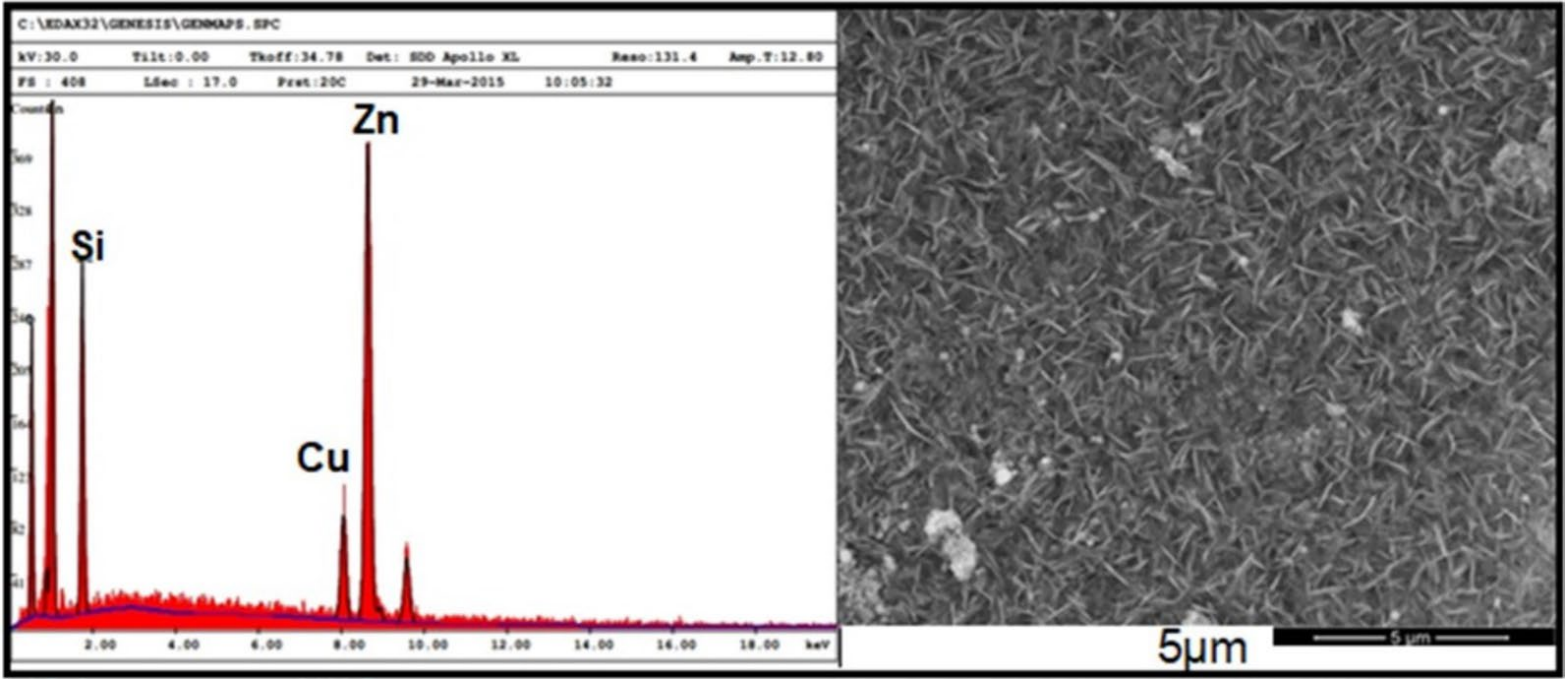
EDX spectrum for Cu-Doped ZnO (11% Cu)

Figure 2 shows the scanning electron micrographs of the morphology of the undoped and Cu-doped ZnO samples. The doped films were deposited under various additions of Cu; 5%, 7%, 9%, 11% and 15% wt, while fixing the molarity of copper nitrate at15µM. From this point on, and for the sake of simplicity, the percentage values refer always to the level of Cu doping in ZnO in wt. %. The film topography is smooth, dusky, uniform, with varying morphology according to Cu-doping. Furthermore, the films strongly adhere to the porous PSi substrates. The surface morphology of the doped Cu–ZnO thin films shows a network of flake-like nanostructures and small clusters of Cu particles at 5% and 7% of Cu-doping, respectively, which agrees with reported results [36]. Formation of nanoplates is observed at 11% and 15% of Cu-doping [37]. The undoped ZnO sheets are aligned very nearly normally to the PSi substrate while doped Cu–ZnO nanostructures are closely fitted to the nano scale of the copper particles found on the surface of the ZnO sheets. The average diameter of the Cu nanoparticles is approximately between 25–75 nm. As the doping of Cu is increased above 9%, the flakes become denser with a continuous-like structure, as shown in Fig. 2 samples (11% and 15% Cu). The concentration of Cu is observed to have a significant effect on the morphology, shape, and size of the films. Figure 3 shows the energy-dispersive X-ray spectroscopy (EDX) image for the pure and the Cu-doped ZnO thin films (11% Cu), which indicates that the as-grown film structures have the Cu–ZnO phase.

**Fig. 4:**
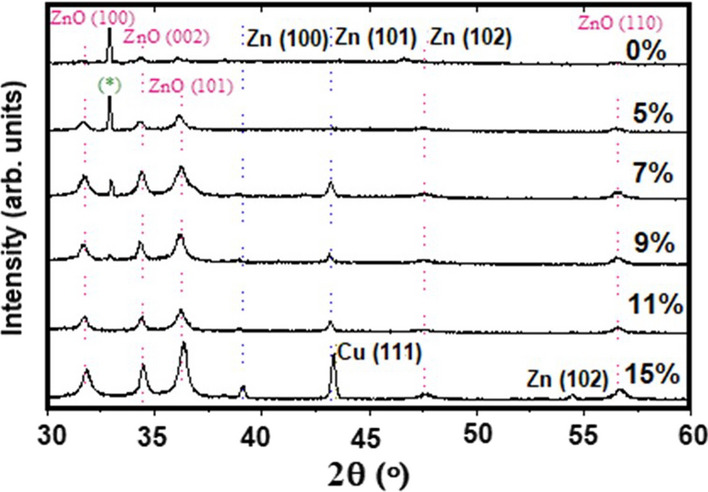
XRD patterns for ZnO films grown at various Cu- doping levels; Top pattern is for pure sample while the bottom one is for the highest doped with 15% of Cu. (*) denotes the contribution from the PSi substrate

The X-ray diffraction analyses were performed to investigate the crystalline microstructure of the deposited films. The typical XRD patterns for the ZnO nanostructures with varying Cu-doping deposited on PSi (100) using ECD technique are shown in Fig. 4. The diffractograms reveal several peaks in Bragg’s angle interval between 30° and 60°. These peaks correspond to reflections from the planes (100), (002), (101), (102), (200) and (110) wurtzite hexagonal (zincite) crystallographic planes, respectively. They show very good agreement with the reported values given in the standard codes (JCPDS No. 36–1451) of ZnO. It is well observed that all doped samples exhibit a preferred growth orientation in the [101] direction.

In all cases, the diffraction peaks observed in Fig. 4 belong to the standard hexagonal wurtzite ZnO structure, which means that the Cu-doping process did not substantially change the original structure. The film exhibits good crystallinity, with narrow peak widths indicating a typical crystallite size in the order of 20–30 nm. All samples showed similar XRD patterns, which is indicative of highly crystalline ZnO hexagonal wurtzite structures.

The peaks are shifted to higher angles and the intensities are increased when increasing the Cu-doping to 15%, compared to that of pure bulk ZnO. This shift in peak positions clearly reflects that Cu replaces Zn in the ZnO films and indicates a change in the strained crystals. In addition, for the 15% Cu-doped sample, three other peaks corresponding to Zn were found at 2θ = 39.0º, 43.3º and 54.3º. This can be attributed to the presence of a high percentage of doping Cu atoms in the ZnO lattice [38–42]. Additionally, for the highest levels of Cu-doping, the former peak attributed to Zn at 43.3º has also contributions from Cu (111), due to its asymmetry.

The lattice parameters (*a* and *c*) present in Table 1 were derived from the (100), (002) and (101) ZnO peak positions in the XRD patterns of Fig. 4. In addition to that, an approximate average size, D, for the Cu–ZnO crystallites was calculated from Scherrer formula [43]:

5 $$ D = \frac{K\lambda }{{\beta \cos \theta }} $$

where *K* is constant and referred to crystal shape and equal to 0.9, *λ* is the incident wavelength of the X-ray (1.5406 Å) and *β* is the FWHM in radians. The values of *D* for all doped samples are listed in Table 1.

**Table 1:** Lattice parameter, crystallite size, peak width, and strain variation for undoped and Cu-doped films

Cu ratio	*a* (Å)	*c* (Å)	*d*_*002*_ (Å)	*D* (nm)	*FWHM*	*ε*_*c*_ (%)	*ε*_*a*_ (%)
0%	3.24686	5.20312	2.610	29.7	0.280	0.06	− 0.097
5%	3.24686	5.20312	2.602	26.7	0.298	0.06	− 0.097
7%	3.25549	5.21137	2.606	31.05	0.257	0.218	0.169
9%	3.25674	5.22268	2.611	31.8	0.250	0.436	0.207
11%	3.26192	5.21902	2.610	23.4	0.340	0.365	0.367
15%	3.25488	5.21228	2.606	20.5	0.388	0.236	0.150

**Fig. 5:**
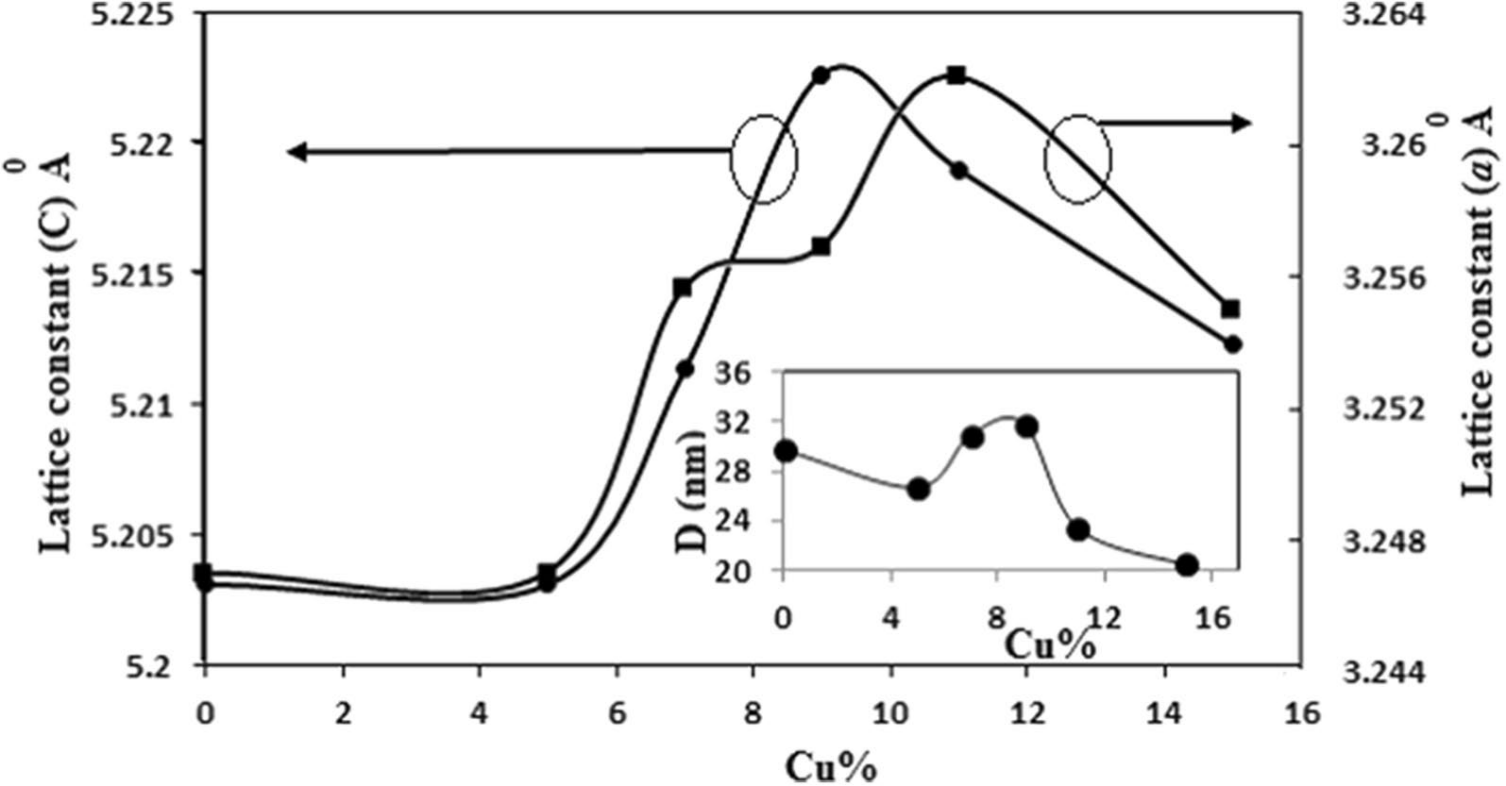
Variation of lattice constants (***a*** and ***c***) and grain size (**D**) for Cu–ZnO with Cu doping concentration

Figure 5 shows the lattice constants and crystallite size as a function of Cu concentration. The lattice constants, *a* and *c* are observed to increase while increasing the Cu-doping concentration in the films until it reaches the maximum value of 9% and decreases above that. The reduction in the lattice parameter values indicates that the Cu^2+^ ions replace Zn^2+^ ions in the Cu–ZnO films. The presence of Cu in the ZnO cell can lead to the reduction of the lattice constants. This observation is consistent with the theoretical results. Theoretically, if most of the Zn atoms are replaced by the Cu atoms, the (002) peak position of Cu–ZnO moves to a slightly higher angle, as compared to that for pure ZnO, since the ionic radius of Cu^2+^ (0.057 nm) is slightly smaller than that of Zn^2+^ (0.060 nm). This is indicative of a compression in the (002) plane spacing that result from the in-plane tensile stress in the cell [44]. Figure 5 also depicts the average crystallite size as a function of Cu concentration, having a maximum value at 9% of Cu-doping and decreasing above this value.

The average crystalline size decreases from 29.7 nm to 26.7 nm when Cu-doping is increased from 0 to 5% and above 9%, indicating that Cu replaces Zn. The decrease in the bond length due to substitution of smaller ionic radii (Cu) with larger ionic radii (Zn) leads to reduction in the lattice parameters and hence, produces compressive stress. The distortion produced around the dopant atom due to the mismatch between ionic radii also reduces the lattice ideality. This small reduction in the lattice dimensions may lead to the decrease of the overall average crystallite size.

The strains ε_*a*_ and ε_c_ which are listed in Table 2 defined by the in-plane (along *a*-axis) and out of plane (along *c*-axis) strains, have been calculated through the relations ε_*a*_ = Δ*a/a*_*o*_ and ε_c_ = Δc/c_o_; where Δ*a* and Δ*c* are defined as the variation of the calculated lattice parameters (*a* and *c*) from the values of the corresponding unstrained bonds in the bulk ZnO material. Positive values for ε_*a*_ and ε_c_ denote tensile strains while negative values denote compressive strains. The positive values of the in-plane strain ε_*a*_ and out of plane strain ε_c_ indicate that the strain caused by the substrate is tensile for all samples except for the two-sample fabricated with 0% and 5% of Cu concentrations.

**Table 2:** The ideality factor, barrier height (BH) and the dark and the photo-current of pure and doped ZnO samples

Sample	Ideality Factor(n)	φ_B0_ (eV)	Current at 3V (A)
Pure ZnO dark	1.5	0.50	3.37 × 10^–3^
Pure ZnO with UV- light	1.2	0.47	5.81 × 10^–3^
Pure ZnO with Visible	1.3	0.48	3.94 × 10^–3^
Doped ZnO dark	2.0	0.49	4.10 × 10^–3^
Doped ZnO with UV-light	1.1	0.32	2.10 × 10^–2^
Doped ZnO with Visible	1.5	0.46	9.70 × 10^–3^

**Fig. 6:**
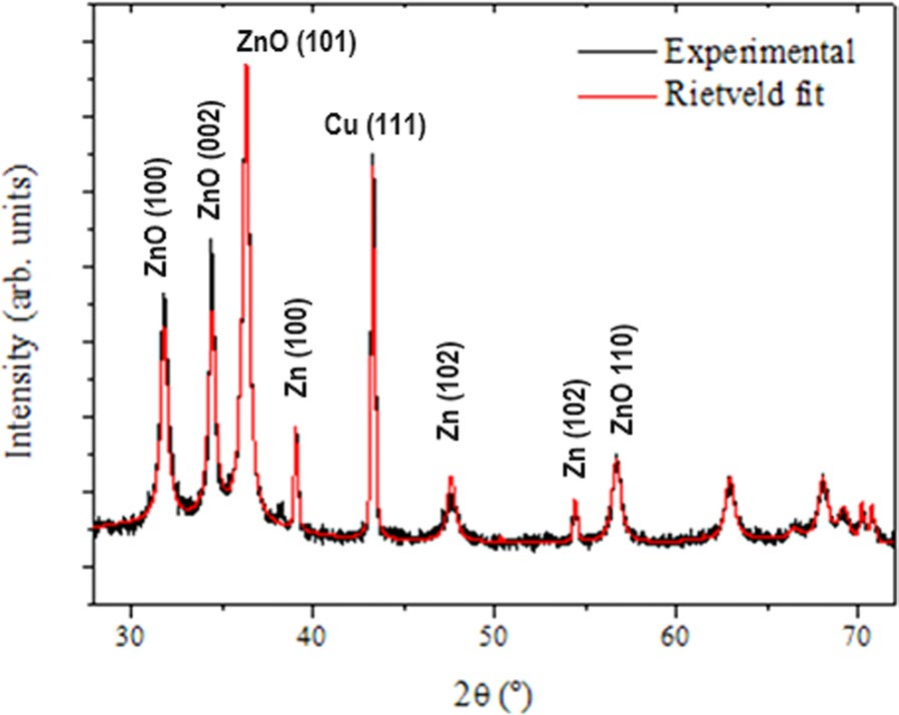
Rietveld refinement to the experimental diffraction pattern of the ZnO film doped with 15% of Cu

Figure 6 displays the Rietveld refinement performed for the diffraction patterns of the heaviest Cu-doped ZnO film (15%). From this refinement, it was possible to determine a quantification of the crystalline phases, consisting of 50% of the zincite wurtzite structure, typical of ZnO, 15% of crystalline Cu and 35% of crystalline zinc. The Cu crystalline grains are very large (~ 250 nm), when compared to those of ZnO and Zn, 36 nm, and 80 nm, respectively. For this sample, the average crystallite size of zincite is slightly higher (23 nm) than that estimated from the Scherrer formula and listed in Table 1 (20.5 nm), due to the fact the Rietveld refinement takes into account domain size from all (hkl) reflections present in the diffraction pattern, unlike the Scherrer formalism which is limited to the analysis of the main peak and has uncertainty in the shape of the crystals. The consumption of zinc into zincite is not optimized in this 15% Cu-doped sample, conversely to what was determined upon the Rietveld refinement of the 11% Cu-doped sample, which revealed 78% of zincite, only 12% of Zn and 10% of Cu. It appears that the excess of Cu promotes entropy in the crystalline growth of the ZnO crystals, where Zn is segregated into individual clusters of grains.

**Fig. 7:**
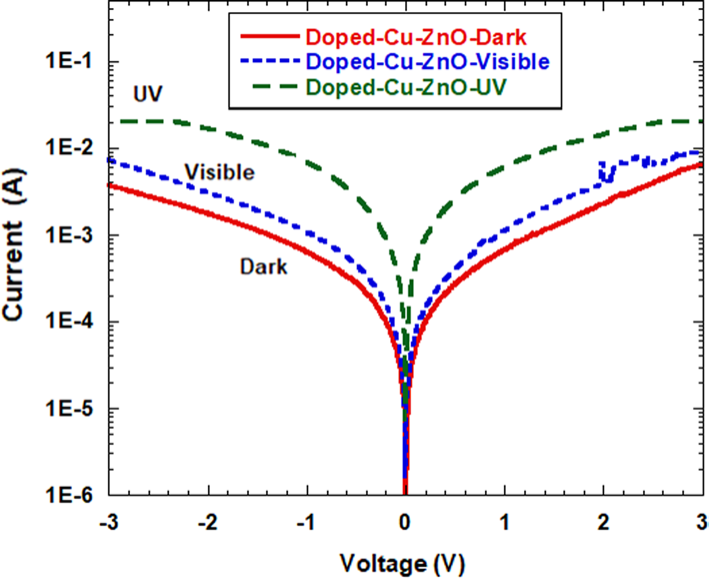
*I-V* characteristics of the fabricated Cu-doped ZnO photodetector measured in dark, visible and under UV illumination

**Fig. 8:**
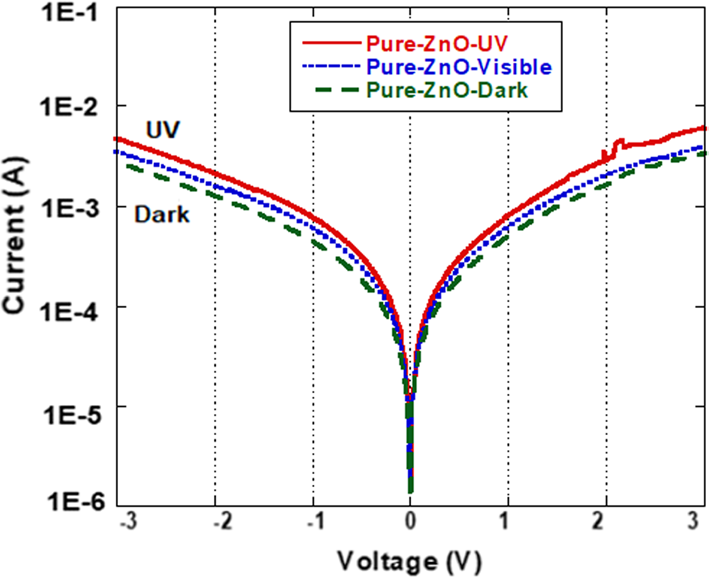
*I-V* characteristics of the fabricated pure ZnO photodetector measured in dark, visible and under UV illumination

Figure 7 and Fig. 8 show the *I*-*V* (current–voltage) characteristics for the photodetector fabricated from Cu-doped ZnO and undoped ZnO materials (with 9% of Cu) measured in dark room and under visible light and UV-illuminations, respectively. The photocurrent under the UV-illumination is obviously higher than that for the dark state in both photodetectors. The Cu-doped photodetector for the dark current exhibits a slight increase in the photocurrent under the whole bias voltage range compared to that for the pure photodetector, whereas the photocurrent becomes larger for the UV and the Visible photodetectors.

The dark current for the doped Cu–ZnO (9% of Cu) sample at 3V bias was found to be 4.1 mA and increases to 21.1 mA under UV illumination. It was found that these *I-V* characteristics can be fitted by the thermionic emission theory as follows [45]:

6 $$ I\, = \,I_{0} \left[ {\exp \left( {\frac{{qV}}{{nkT}}} \right) - 1} \right] $$

where *V* is the applied voltage across the diode, *n* is the ideality factor, *K* is Boltzmann constant and *I*_o_ is the saturation current given by:

7 $$ I_{0} = AA^{**} T^{2} \exp \left[ {\frac{{ - q\varphi_{B0} }}{kT}} \right] $$

where *q* is the electron charge, *T* is the temperature in Kelvin, *A* is the contact area, A** is the effective Richardson constant and *φ*_*B*0_ is the energy barrier height. The ideality factors and the barrier height (BH) were calculated from Eqs. (6) and (7). Table 2 summarizes the dark and the photo-current measured at 3V, as well as the ideality factor and BH for the pure and the Cu-doped ZnO samples found from the *I-V* characteristics.

**Fig. 9:**
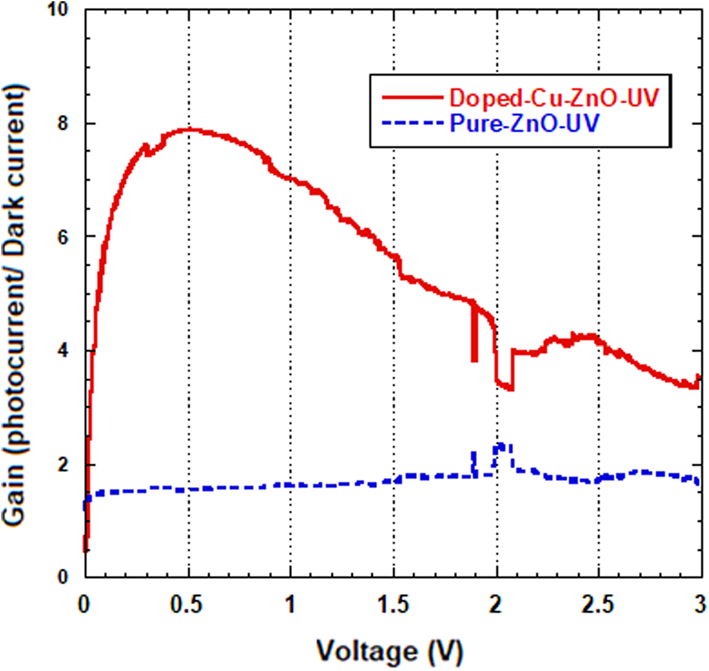
Current gain for the fabricated pure ZnO and doped photodetector for UV/dark as a function of the voltage bias

Schottky barrier height was found to be influenced by both illumination and doping. The barrier heights for both dark and illuminated conditions were observed to be of a higher value for the pure sample when compared to that for the Cu-doped sample. Under illumination conditions, the barrier heights for both pure and doped samples became smaller. This indicates the presence of a higher current in the photodetectors [46]. It should be noted that the variation registered for the barrier height under dark and UV illumination was 0.17 eV for 9% Cu-doped sample, as compared to 0.03 eV for the pure ZnO sample. This means that the doped ZnO sample was more sensitive to UV illumination compared to the non-doped sample. The outcomes found in our study were further investigated by evaluating the contrast ratio of photocurrent and dark current gains, as seen in Fig. 9. The maximum contrast ratio (gain) of the UV to the dark conditions for the Cu- doped ZnO and the pure ZnO photodetectors was found to be 8 and 1.8, respectively. The contrast ratios for the visible and the dark conditions were found to be 1.5 and 1.2, respectively. This reveals that, the sensitivity of the photodetector fabricated based on Cu- doped ZnO is of much higher value compared to that for the pure ZnO photodetector, with values of approximately 5 times for UV illumination, while it is of about 1.25 times for the visible illumination.

The high values of photocurrents found for the Cu-doped ZnO films compared to those found for the undoped ZnO films may be attributed due to the enhancement of their crystallinity, as well as, due the enhancement of the optical transparency in the visible region which may be attributed to the Cu doping effects.

## Conclusions

Pure and Cu–ZnO thin films have been fairly synthesized on porous Si substrates by a low-cost and one-step electrodeposition technique. ZnO films present a pure phase and a crystal size on the nano-metric scale. The film’s surface is smooth, dusky, uniform and strongly adherent to the porous substrate. It was possible to observe different surface morphologies for the grown structures according to the variation of concentrations of the Cu dopant as investigated by the SEM micrographs. Networks of flake-like nanostructures are observed on the surface of the Cu–ZnO thin films as confirmed by the SEM micrographs. From XRD diffraction experiments and subsequent analysis, polycrystalline ZnO with a wurtzite structure and the formation of a Cu–ZnO composite was identified and having a preferential orientation normal to the (101) planes. In addition, the XRD analysis showed the incorporation of the Cu ions through the replacement of the Zn atoms in the ZnO crystal lattice. The lattice constants and the average size of ZnO crystals vary with the Cu-doping concentration. The in-plane strain (ε_*a*_) and the out-of-plane strain (ε_c_) indicate that the strain caused by the substrate is tensile for the doped samples. The UV photodetector fabricated on Cu-doped ZnO films shows highly sensitive response with current gain of about 5 times that obtained from pure one as illustrated from I-V measurements. The spectral response curves for the films integrated as photodetectors show that the films have considerable response to the UV light illumination.

## Data Availability

They are available from the corresponding author upon reasonable request.
